# Supplementary material for: Factors influencing integrated disease surveillance and response system in selected districts in the Eastern Region of Ghana
Source: PLoS One. 2024 Aug 13;19(8):e0295473. doi: 10.1371/journal.pone.0295473 (PMC11321551; doi:10.1371/journal.pone.0295473)
Supplement: S1 Text — (DOCX) [file pone.0295473.s001.docx]

**Questionnaire**

**Participants information sheet**

This information sheet is to inform the study participants about the research for them to make an informed decision of whether to participate in the study or not. It also outlines the nature of the research, what the research involves, risks, benefits, and compensation.

**Title of Study:**

“Factors Influencing Integrated Disease Surveillance and Response System in Selected Districts in Eastern Region of Ghana”

**Introduction:**

The field supervisor for this study is Paul Twene**,** a health worker at the Eastern Regional Health Directorate. His email address is [twenepaul12@gmail.com](mailto:twenepaul12@gmail.com) and telephone number is 0203087128. The academic supervisor for this study is Dr. Bismark Sarfo (Email: [bsarfo@ug.edu.gh](mailto:bsarfo@ug.edu.gh), Tel: 0244244892)

**Background and Purpose of Research:**

The research topic for this study is “Factors Influencing Integrated Disease Surveillance and Response System in Selected Districts in the Eastern Region of Ghana”.

**Nature of Research:**

The research is a cross-sectional study with a quantitative approach. My interest is in finding out the factors influencing Integrated Disease Surveillance and Response System in selected Districts in Eastern Region of Ghana. It would be conducted among health workers who are actively involved in disease surveillance activities in the New Juaben South Municipality, Abuakwa South Municipality, and Fanteakwa North District in the Eastern Region of Ghana.

**PARTICIPANTS INVOLVEMENT**

**Duration/ what is involved:**

A structured questionnaire would be used to elicit information from the study participants after the aim of the study has been explained to them and they are interested in participating. The questionnaire would be administered in the English Language for the respondents. This would last for approximately 10 minutes.

**Potential Risks:**

There is potential risk of contracting COVID-19 under the current circumstances of the COVID-19 pandemic. Therefore, the following interventions would be put in place to ensure the safety of the study participants and the research assistants;

- The researcher and assistants will be in personal protective equipment that includes face mask and face shield.
- Participants will wash their hands with soap under running water as observed by the research assistants before receiving the questionnaire
- Participants will sanitize their hands with alcohol-based rubs after filling the questionnaires
- Social distancing will be maintained between the participants and the research assistants

**Benefits:**

Participants would have the opportunity to gain some knowledge on the factors that contribute influence IDSR since each participant would be educated after data collection.

**Cost:**

There would be no cost incurred by participants for taking part of the study except their time.

**Compensation:**

There would not be any compensation for participating in the study.

**Confidentiality:**

In ensuring anonymity, participants would only be identified with codes and numbers. No information regarding participants name or any other information that traces the data collected to the participants would be taken. Filled questionnaires would be kept under lock and key, with only the principal investigator having access.

**Voluntary participation/ withdrawal:**

Participation in the study is voluntary and not compulsory. Participants have the right to decide whether or not they want to be part of the study. You can also withdraw your consent at any time of the study.

**Outcome and feedback:**

Findings of the study will be shared with the selected districts and Eastern Regional Health Directorate to help in making informed decisions pertaining to surveillance activities in the region.

**Feedback to participants:**

A report would be presented to various stakeholders such as the Ministry of Health (MOH), Ghana Health Service and the selected districts to help them in their activities. The report will be published in a journal.

**Funding information:**

This study is funded by the Principal Investigator.

**Sharing of Participants Information/Data:**

Participant information or data would be kept by principal investigator. The uploaded administered questionnaires from the web based would be kept under kept securely with a password, with only the principal investigator having access. It would not be shared with anyone else.

**Provision of Information and Consent for participants:**

A copy of the information sheet and consent form will be given to the participants to sign or thumb-print before participation in the study

**Who to Contact for Further Clarification/Questions:**

For any clarifications or concerns concerning this research, please contact Paul Twene at the Eastern Regional Health Directorate on telephone number 0203087128 or by e-mail at [twenepaul12@gmail.com](mailto:twenepaul12@gmail.com). For further clarification on ethical issues kindly contact the Ghana Health Service Ethical Review Committee Administrator, Nana Abena Apatu on phone number 0503539896.

**Appendix II: Consent form**

**STUDTY TITLE:** Factors Influencing Integrated Disease Surveillance and Response System in Selected Districts in the Eastern Region of Ghana”.

**PART I: PARTICIPANTS’ CONSENT FORM/ CERTIFICATE OF CONSENT**

I have been invited to participate in research on “Factors Influencing Integrated Disease Surveillance and Response System in Selected Districts in Eastern Region of Ghana”.

The document describing the nature and purpose as well as risks and benefits of the study has been read and explained to me. I have been allowed to have any questions about the study answered to my satisfaction. I agree voluntarily to participate in this study.

**_______________________ _________________ __________**

Full name of participant Signature or Thumbprint Date

**DECLARATION BY WITNESS (IF PARTICIPANT CANNOT READ BY HIM/HERSELF)**

I was present while the benefits, risks and nature, and purpose of the study were read to the participant. All questions were answered and the participant has agreed voluntarily to take part in the study.

**__________________ _________________ __________**

Full name of participant Signature or Thumb print Date

**RESEARCHERS SIGNATURE COLUMN**

I certify that the nature and purpose, the potential benefits, and possible risks associated with participating in this study have been explained to the above individual to the best of my ability. I confirm that the participant was given an opportunity to ask questions about the study, and all the questions asked by the participant have been answered correctly and to the best of my ability. I confirm that the consent has been given freely and voluntarily.

_______________________ __________________________ ____________________

Name of researcher Signature Date

**Appendix III: Questionnaire**

| SN | QUESTION | RESPONSE |
| --- | --- | --- |
| BACKGROUND CHARACTERISTICS OF RESPONDENT | | |
| 1 | Facility type | 1. Hospital 2. Health Centre 3. CHPS 4. Clinic 5. Maternity |
| 2 | Duration at current position (in years) | 1. Less than 1 year 2. 1 Year 3. 2 Years 4. More than three years |
| 3 | Age of respondent |  |
| 4 | Educational qualification | 1. None 2. Primary 3. JHS 4. SHS/SSS 5. Vocational/Technical 6. Tertiary |
| 5 | Cadre/Category of staff | 1. Disease Control Officer 2. Medical Officer 3. Community Health Nurse 4. Midwife 5. Enrolled Nurse 6. Physician Assistant 7. Records Officer 8. Others (Specify) |
| 6 | Availability of staff for surveillance activities | 1. Yes 2. No |
| 7 | How may staffs are available for surveillance activities at this facility? |  |
| AVAILABILITY OF DATA COLLECTION TOOLS AND LOGISTICS FOR SURVEILLANCE ACTIVITIES | | |
| 8 | Do you have a register for recording/logging rumors? | 1. Yes 2. No |
| 9 | Do you have IDSR technical guidelines? | 1. Yes 2. No |
| 10 | Do you have the following forms for IDSR? | |
|  | 1. Immediate reporting forms | 1. Yes 2. No |
|  | 1. Weekly reporting forms | 1. Yes 2. No |
|  | 1. Monthly reporting forms | 1. Yes 2. No |
|  | 1. Case based form for AFP | 1. Yes 2. No |
|  | 1. Case based forms for measles | 1. Yes 2. No |
|  | 1. Case based form for meningitis | 1. Yes 2. No |
|  | 1. Yellow fever case-based form | 1. Yes 2. No |
|  | 1. Case based form for COVID-19 | 1. Yes 2. No |
|  | 1. Availability of generic case-based form | 1. Yes 2. No |
| 11 | Is this facility equipped with either a tablet or computer for surveillance data management | 1. Yes 2. No |
| 12 | Availability of specimen carriers for transporting samples | 1. Yes 2. No |
| 13 | Availability of sample containers for blood or serum samples | 1. Yes 2. No |
| 14 | Availability of sample containers for AFP stool samples | 1. Yes 2. No |
| 15 | Availability of sample containers for cerebrospinal fluid collection | 1. Yes 2. No |
| 16 | Availability of sample containers for sputum samples | 1. Yes 2. Yes |
| 17 | Do you have means of transport for surveillance activities | 1. Yes 2. No |
| CORE SURVEILLANCE FUNCTIONS | | |
|  | **CASE DETECTION AND REGISTRATION** |  |
| 18 | Do you have consulting room register? | 1. Yes 2. No |
| 19 | Do you have standard case definitions for the priority diseases? | 1. Yes 2. No |
| 20 | Do you conduct records review at this facility | 1. Yes 2. No |
| 21 | How often do you conduct records review at this facility? | 1. Yes 2. No |
| 22 | Observe each of the staff involved in surveillance activities correctly applying the standard case definition for detection of priority diseases (use at least two of the priority diseases as a proxy) | 1. Yes 2. No |
|  | **CASE CONFIRMATION** | |
| 23 | Is this facility capacitated to collect each of the following specimen? | |
|  | 1. Sputum | 1. Yes 2. No |
|  | 1. Stool | 1. Yes 2. No |
|  | 1. Blood | 1. Yes 2. No |
|  | 1. Cerebrospinal fluid (CSF) | 1. Yes 2. No |
| 24 | Is facility having the capacity to handle sputum, stool, blood, and CSF until shipment at this facility? | 1. Yes 2. No |
| 25 | Do you have packing materials for shipment of specimens at the health facility? (Observe to confirm) | 1. Yes 2. No |
| 26 | How long does it take for samples to be transported to the region? | 1. Less than 24 hours 2. Within 24 hours 3. Within 48 hours 4. Within 72 hours 5. More than 72 hours |
| 27 | Have you lacked weekly IDSR reporting form during the last 6 months? | 1. Yes 2. No |
| 28 | Is this facility in the position to process samples for laboratory investigation | 1. Yes 2. No |
| 29 | Are the laboratory services readily available to assist in samples processing | 1. Yes 2. No |
| DATA REPORTING | | |
| 30 | Number of weekly IDSR reports submitted in the last 3 months? | |
| 31 | Number of monthly reports submitted in the last 3 months |  |
| 32 | By what means do you report to the next higher level? | 1. Yes 2. No |
|  | **DATA ANALYSIS (Observe)** | |
| 33 | Availability of data analyzed by person, place and time | 1. Yes 2. No |
|  | **RESPONSE** | |
| 34 | Is this facility resourced to implement control and prevention measures against local disease? | 1. Yes 2. No |
| 35 | How long does it take for higher levels to respond when your surveillance needs? | 1. Less than 24 hours 2. Within 24 hours 3. Within 48 hours 4. Within 72 hours 5. More than 72 hours |
| 36 | Do you have thresholds for epidemic prone diseases at this facility | 1. Yes 2. No |
| 37 | Do you have epidemic preparedness plan for the epidemic prone diseases | 1. Yes 2. No |
| 38 | Please indicate which of the epidemic prone diseases has an epidemic preparedness plan available |  |
| 39 | Do you have rapid response team at this facility | 1. Yes 2. No |
| 40 | If yes, do you have evidence of minutes of their meetings | 1. Yes 2. No |
|  | **FEEDBACK** | |
| 41 | Do you receive feedback on surveillance from higher levels? | 1. Yes 2. No |
| 42 | How many feedback reports have you received in the last year? |  |
| 43 | How many meetings have you conducted with the community in the past year to discuss surveillance related issues? |  |
| 44 | Do you discuss surveillance related activities at during your review meetings? | 1. Yes 2. No |

**
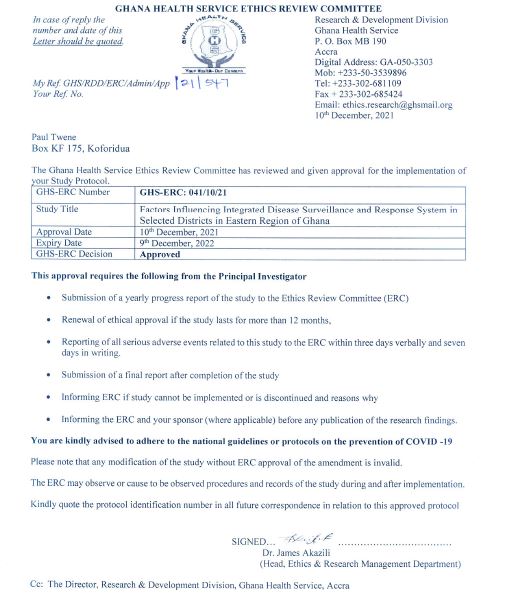
Appendix IV: Ethical clearance letter**
